# Hydrostatin-SN1, a Sea Snake-Derived Bioactive Peptide, Reduces Inflammation in a Mouse Model of Acute Lung Injury

**DOI:** 10.3389/fphar.2017.00246

**Published:** 2017-05-05

**Authors:** Guosheng Wu, Junjie Wang, Pengfei Luo, An Li, Song Tian, Hailong Jiang, Yongjun Zheng, Feng Zhu, Yiming Lu, Zhaofan Xia

**Affiliations:** ^1^Department of Burn Surgery, Changhai Hospital, Second Military Medical UniversityShanghai, China; ^2^Department of Biochemical Pharmacy, School of Pharmacy, Second Military Medical UniversityShanghai, China

**Keywords:** acute lung injury, hydrostatin-SN1, lipopolysaccharide, inflammation, ERK1/2, NF-κB

## Abstract

Snake venom has been used for centuries as a traditional Chinese medicine. Hydrostatin-SN1 (H-SN1), a bioactive peptide extracted from the *Hydrophis cyanocinctus* venom gland T7 phage display library, was reported to have the ability to reduce inflammation in a dextran sulfate sodium-induced murine colitis model. In this study, we sought to investigate the inhibitory potential of H-SN1 on inflammation in a murine model of lipopolysaccharide (LPS)-induced acute lung injury (ALI), and elucidate the anti-inflammatory mechanism in LPS-stimulated RAW 264.7 cells. *In vivo*, C57BL/6 male mice were intratracheally instilled with LPS or physiological saline with concurrent intraperitoneal injection of H-SN1 or saline alone. Lung histopathologic changes, lung wet-to-dry weight ratio, and myeloperoxidase activity in lung tissues were assessed. Total cell number, the protein concentration, and cytokine levels were determined in the bronchial alveolar lavage fluid. *In vitro*, RAW 264.7 cells were treated with various concentrations of H-SN1 for 2 h followed by incubation with or without 1 μg/ml LPS for 0.5 or 24 h. The mRNA expression of inflammatory cytokines was determined via RT-PCR and protein levels in the supernatants were measured via ELISA. Extracellular-signal related kinase 1/2 (ERK1/2) and nuclear factor-κB (NF-κB) pathways were analyzed via western blot. H-SN1 improved pulmonary edema status, decreased vascular permeability, suppressed pro-inflammatory cytokine production, and lessened lung morphological injury. H-SN1 also dose-dependently inhibited the mRNA expression and release of TNF-α, IL-6, and IL-1β in LPS-stimulated RAW 264.7 cells. Moreover, H-SN1 inhibited the LPS-induced phosphorylation of ERK1/2 and the nuclear translocation of NF-κB. Our results suggest that H-SN1 could attenuate LPS-induced ALI in mice, which is associated with the anti-inflammatory effect of H-SN1. The mechanism might involve inhibiting the production of inflammatory cytokines by, at least in part, interfering with the ERK1/2 and NF-κB signaling pathways.

## Introduction

Acute lung injury (ALI), a life-threatening disease, is characterized by severe hypoxemia, pulmonary edema, and neutrophil accumulation in the lung (Ware and Matthay, 2000). Intratracheal administration of lipopolysaccharide (LPS), the main component of gram-negative bacterial walls, is a widely accepted experimental model of ALI (Kitamura et al., 2001; Wu et al., 2002), as LPS stimulates profound lung recruitment of inflammatory cells and the subsequent increase in pro-inflammatory cytokines. Macrophages are important immune cells that mediate the initiation of inflammatory reactions by producing multiple pro-inflammatory cytokines and enzymes in response to various stimuli, including LPS (Laskin and Pendino, 1995; Huang et al., 2014). Those pro-inflammatory mediators include tumor necrosis factor alpha (TNF-α), interleukin-6 (IL-6), interleukin-1β (IL-1β), cyclooxygenase-2, and metalloproteinases, which are involved in the pathogenesis of ALI. Previous studies have demonstrated that anti-inflammatory compounds are beneficial in treating LPS-induced lung injury in mice (Chen et al., 2008; Zhong et al., 2013; Qi et al., 2014).

Hydrostatin-SN1 (H-SN1) is a bioactive tumor necrosis factor receptor 1 (TNFR1)-binding peptide recently screened from the *Hydrophis cyanocinctus* venom gland T7 phage display library. H-SN1 has been reported to reduce inflammation in a dextran sulfate sodium-induced murine colitis model (Zheng et al., 2016). Furthermore, H-SN1 can inhibit TNF-α-mediated activation of the nuclear factor-kappa B (NF-κB) and mitogen-activated protein kinase (MAPK) signal pathways.

In the present study, we sought to investigate the inhibitory potential of H-SN1 on inflammation in the LPS-induced mouse ALI model, and elucidate the anti-inflammatory mechanism of H-SN1 in LPS-stimulated RAW 264.7 cells. In our study, we found that H-SN1 treatment attenuated the inflammatory response in LPS-induced ALI, and its protective effect might involve the inhibition of the production of inflammatory mediators such as TNF-α, IL-6, and IL-1β, partially by interfering with the ERK1/2 and NF-κB signaling pathways.

## Materials and Methods

### Regents

Hydrostatin-SN1 was kindly provided by School of Pharmacy, Second Military Medical University, Shanghai, China. LPS was purchased from Sigma (St. Louis, MO, USA). Cell culture reagents were purchased from Invitrogen (Carlsbad, CA, USA). Enzyme-linked immunosorbent assay (ELISA) kits of TNF-α, IL-6, and IL-1β were obtained from R&D Systems (Minneapolis, MN, USA). The ECL Chemiluminescence kit and bicinchoninic acid (BCA) protein assay were purchased from Thermo Fisher Scientific Inc. (Waltham, MA, USA). The rabbit monoclonal antibodies for extracellular-signal related kinase 1/2 (ERK1/2), phospho-ERK1/2, NF-κBp65, phospho-NF-κBp65, IκBα and mouse monoclonal antibody for GAPDH were purchased from Cell Signaling Technology Inc. (Beverly, MA, USA). The horseradish peroxidase-conjugated goat anti-rabbit and goat anti-mouse secondary antibodies were provided by Santa Cruz Biotechnology (Dallas, TX, USA). Trizol reagent was purchased from Invitrogen (Carlsbad, CA, USA). Red blood cell lysis buffer and myeloperoxidase (MPO) assays were purchased from Beyotime Institute of Biotechnology (Jiangsu, China).

### Animals and Cell Culture

Male C57BL/6 mice, weighing 20–25 g, were purchased from Experimental Animal Center, Second Military Medical University (Shanghai, China). Mice were housed in individual cages in controlled conditions (23 ± 3°C, 50 ± 10% humidity, and 12 h day/night cycle) with free access to food and water. All animal experiments were conducted according to the Guide for the Care and Use of Laboratory Animals published by the National Institutes of Health, and the protocol was approved by the Animal Care and Use Committee of the Second Military Medical University.

The murine macrophage cell line RAW 264.7 was purchased from ATCC. The cell line was cultured in Dulbecco’s Modified Eagles Medium (DMEM) with 10% FBS at 37°C under a humidified atmosphere of 5% CO2.

### Model of LPS-Induced ALI

The LPS-induced ALI model was performed as previously described (Peng et al., 2004). Thirty-two mice were randomly divided into four groups (*n* = 8 in each group): (1) control group, mice were instilled with 50 μl physiological saline intratracheally via a 20-gauge catheter; (2) H-SN1 group, H-SN1 was dissolved in physiological saline at a concentration of 5 mg/ml and injected (i.p., 400 μg/kg); (3) LPS group, 2 mg/kg LPS diluted in 50 μl physiological saline was instilled intratracheally; and (4) LSP + H-SN1 group, H-SN1 was dissolved in physiological saline with a concentration of 5 mg/ml and administered (i.p., 400 μg/kg) 60 min before LPS instillation. The H-SN1 dose was determined in preliminary experiments in which mice were administered 200, 400, 600, and 800 μg/kg H-SN1; the 400 μg/kg dose was the minimum required to achieve the greatest attenuation of inflammation. Four mice of each group were taken for bronchial alveolar lavage (BAL) fluid analysis. Twenty-four hours after LPS challenge, BAL was collected by instilling and withdrawing sterile physiological saline (0.8 ml) through a tracheal cannula using a 20-gauge catheter three times. The three BAL fluid samples were pooled then centrifuged (4°C, 1000 × *g*/min, 5 min), and the supernatants were stored at -80°C until further examination. After that, the right lobes were isolated to extract RNA and the left lobes were used for MPO activity assay and extracting protein.

### Histopathology

The right lobes of the remaining four mice were fixed in 10% formaldehyde, embedded in paraffin, cut into 5-μm-thick sections and stained with hematoxylin and eosin (H&E).

### Lung Wet-to-Dry Weight Ratio (W/D)

The whole left lobes of the remaining four mice were dissected free of surrounding connective tissue. After being weighed, the lungs were dried in an oven at 70°C for 48 h and reweighed.

### MPO Activity Assay

Myeloperoxidase activity was determined as previous reported (Sun et al., 2015). A part of left lung tissues was homogenized and processed, and MPO activity was measured according to the manufacturer’s instructions.

### Cell Counting and Protein Concentration in BAL Fluids

After centrifugation of BAL fluid, erythrocytes in the cell pellets were lysed using red blood cell lysis buffer and the remaining cells were resuspended in PBS for the total cell counts using a hemocytometer. The total protein concentration of BAL fluid was determined using Lowry’s method.

### Quantitative Real-Time PCR

RAW 264.7 cells were plated onto 12-well plates (10^5^ cells/well) and treated with different concentrations of H-SN1 (1, 5, and 10 μM) for 2 h, followed by incubation with or without 1 μg/ml LPS for 3 and 6 h. Then, the supernatant was collected for measurement of cytokines, and total RNA was extracted from cells using Trizol. Quantitative real-time PCR was carried out using SYBR Green PCR Master Mix in a total volume of 10 μl on Step One Plus Real-Time PCR System (Applied Biosystems). Primer sequences were as follows: GAPDH (FP: AGG TCG GTG TGA ACG GAT TTG; RP: TGT AGA CCA TGT AGT TGA GGT CA), TNF-a (FP: ACT CTG ACC CCT TTA CTC TG; RP: GAG CCA TAA TCC CCT TTC TA), IL-6 (FP: CCA ATG CTC TCC TAA CAG AT; RP: TGT CCA CAA ACT GAT ATG CT), IL-1β (FP: TTC AGG CAG GCA GTA TCA 3; RP: GTC ACA CAC CAG CAG GTT AT).

### Cytokine Assays

The concentrations of cytokines TNF-α, IL-6, and IL-1β in the BAL fluid and cell supernatants were measured using mouse ELISA kits, according to the manufacturer’s instructions.

### Western Blot Analysis

In a separate set of experiments, RAW 264.7 cells were plated onto 6-well plates (10^6^ cells/well) and treated with 0 or 10 μM H-SN1 for 2 h, followed by stimulation with or without 1 μg/ml LPS for 30 min. Then, the cells were lysed in lysis buffer after washing (×2) with ice-cold PBS buffer. The extract was centrifuged (4°C, 1500 rpm/min, 10 min) and the supernatant was collected. Protein concentration was determined using the BCA protein assay kit. Western blot analysis was performed with ERK1/2, phospho-ERK1/2, NF-κBp65, phospho-NF-κBp65, IκBα and GAPDH monoclonal antibodies as previously described (Huang et al., 2014).

### Statistical Analysis

Each experiment was conducted at least three independent times. All values are expressed as means ± standard error of the mean (SEM). Differences between groups were analyzed using one-way analysis of variance (Dunnett’s *t*-test) and two-tailed Student *t*-test. *P* < 0.05 or *P* < 0.01 was considered statistically significant.

## Results

### *In Vivo* Experiment

#### H-SN1 Alleviates LPS-Induced Lung Histopathological Damages

**FIGURE 1:**
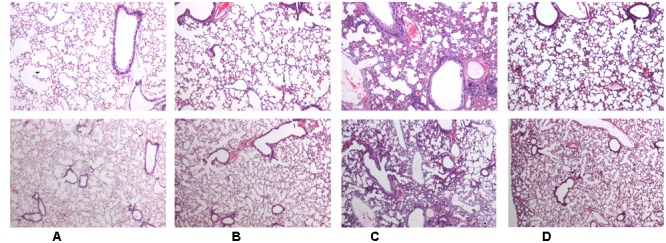
**Effects of hydrostatin-SN1 (H-SN1) on histopathological changes in lung tissues of lipopolysaccharide (LPS)-induced acute lung injury (ALI) mice. (A)** Control mice; **(B)** H-SN1 treated mice; **(C)** LPS treated mice; and **(D)** LPS + 400 μg/kg H-SN1 treated mice. The lung sections from different groups were stained with hematoxylin and eosin (H&E) solution (Upper panels: magnification ×50, Bottom panels: magnification ×100). Raw data can be seen in **Supplementary Figures S1A–D**.

Twenty-four hours after LPS instillation, histological examination of the lung sections revealed significant infiltration of inflammatory cells, diffuse alveolar hemorrhage, and large amounts of inflammatory exudates compared to those in the mice in the control and H-SN1 groups. Importantly, the severity of ALI as assessed by histopathological evaluation was markedly alleviated by H-SN1 treatment (**Figure 1**; Raw data can be seen in **Supplementary Figures S1A–D**).

#### H-SN1 Decreases LPS-Induced Lung Vascular Permeability

**FIGURE 2:**
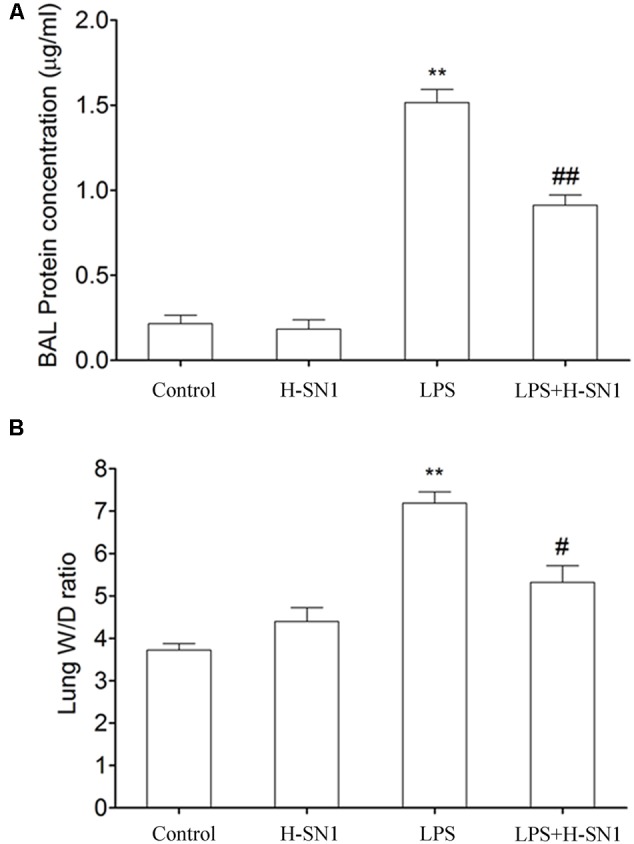
**Effects of H-SN1 on lung vascular permeability of LPS-induced ALI mice.** Bronchial alveolar lavage (BAL) fluids protein concentrations **(A)** and lung W/D ratio **(B)** were measured at 24 h post injury. Data were presented as mean ± SEM (*n* = 3∼4 in each group), ^∗∗^*p* < 0.01 vs. Control group, ^#^*p* < 0.05 and ^##^*p* < 0.01 vs. LPS group.

Increased BAL fluid protein concentrations and lung W/D ratios are two common indicators of pulmonary vascular permeability. Thus, we investigated the effect of H-SN1 on these parameters. Both BAL fluid protein concentrations (**Figure 2A**) and the lung W/D ratio (**Figure 2B**) markedly increased 24 h after LPS instillation compared to those in the control and H-SN1 groups. In contrast, H-SN1 treatment significantly decreased BAL fluid protein concentrations (*P* < 0.01) and the lung W/D ratio (*P* < 0.05). These data indicated that H-SN1 treatment could attenuate lung vascular permeability induced by LPS.

#### H-SN1 Reduces Total Cells in BAL Fluids of LPS-Induced ALI

**FIGURE 3:**
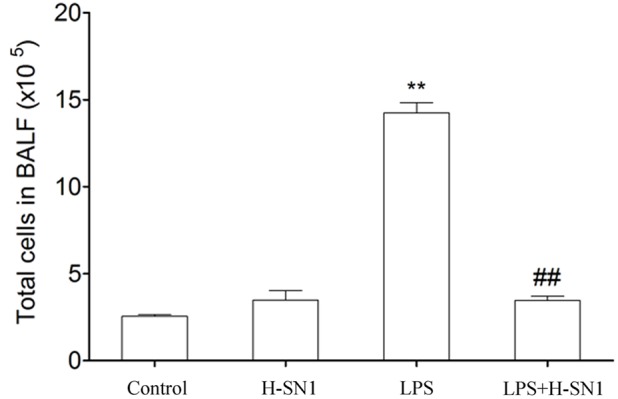
**Effect of H-SN1 on the number of total cells in BAL fluids of LPS-induced ALI mice.** Data were presented as mean ± SEM (*n* = 3∼4 in each group), ^∗∗^*p* < 0.01 vs. Control group, ^##^*p* < 0.01 vs. LPS group.

Total cells in BAL fluid were also analyzed. Compared to the control and H-SN1 groups, total cells were significantly increased in the lung alveoli of mice instilled with LPS alone (**Figure 3**, *P* < 0.01) and this increase was markedly reduced by H-SN1 treatment.

#### H-SN1 Decreases MPO Activity in LPS-Induced ALI

**FIGURE 4:**
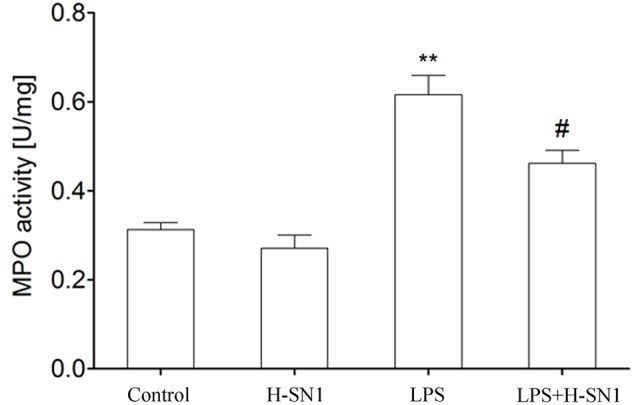
**Effect of H-SN1 on myeloperoxidase (MPO) activity in the lungs of LPS-induced ALI mice.** Data were presented as mean ± SEM (*n* = 4 in each group), ^∗∗^*p* < 0.01 vs. Control group, ^#^*p* < 0.05 vs. LPS group.

Myeloperoxidase contributes to the development of LPS-induced ALI and MPO activity is an indicator of neutrophil accumulation. Our results showed that LPS challenge caused a significant upregulation of MPO activity in lung tissues. H-SN1 treatment significantly decreased the MPO activity compared to LPS group (**Figure 4**, *P* < 0.05).

#### H-SN1 Inhibits Cytokines Production in BAL Fluids of LPS-Induced ALI

**FIGURE 5:**
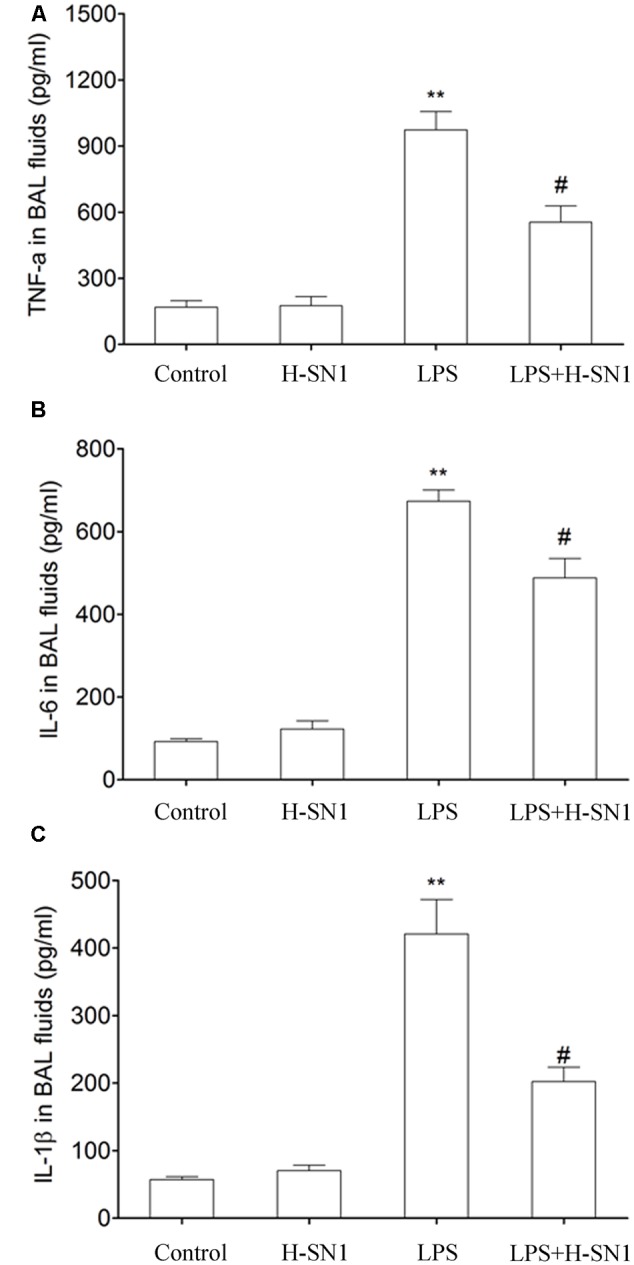
**Effect of H-SN1 on cytokines production in BAL fluids of LPS-induced ALI mice.** Tumor necrosis factor alpha (TNF-α) **(A)**, interleukin-6 (IL-6) **(B)**, and interleukin-1β (IL-1β) **(C)** levels in BAL fluids were measured at 24 h post injury. Data were presented as mean ± SEM (*n* = 3∼4 in each group), ^∗∗^*p* < 0.01 vs. Control group, ^#^*p* < 0.05 vs. LPS group.

Cytokines such as TNF-α, IL-6, and IL-1β are critical mediators of inflammation. In this study, we investigated the effect of H-SN1 on the production of these inflammatory cytokines in the BAL fluid of different groups. There were significantly elevated levels of TNF-α, IL-6, and IL-1β in BAL fluid from LPS-treated mice compared to control and H-SN1 groups (*P* < 0.01) (**Figure 5**). Notably, these increases were attenuated by H-SN1 (*P* < 0.05), suggesting the potential anti-inflammatory effect of H-SN1.

#### H-SN1 Inhibits NF-κB Activation and ERK Activation in LPS-Induced ALI

**FIGURE 6:**
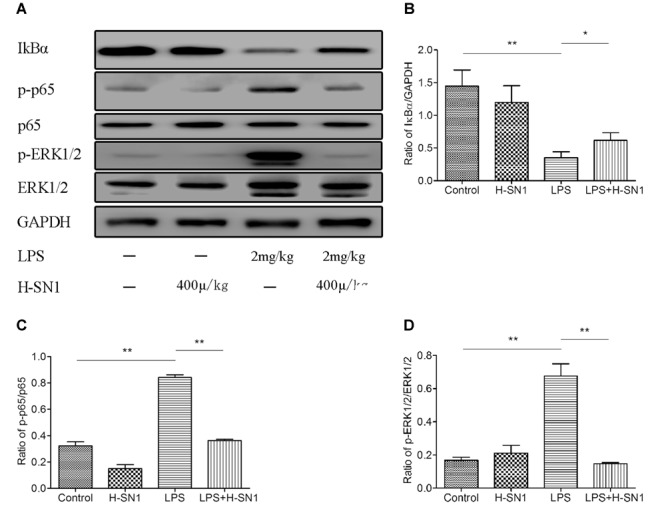
**Effect of H-SN1 on LPS-induced nuclear factor-κB (NF-κB) and mitogen-activated protein kinase (MAPK) signaling activation in lung tissues.** The phosphorylation of NF-κBp65 and extracellular-signal related kinase ½ (ERK1/2) proteins were analyzed by Western blot. **(A)** A representative immunoblot from three separate experiments with similar results is shown. **(B–D)** Relative protein levels were quantified by ImageJ software and expressed as optical density ratio. ^∗^*p* < 0.05 and ^∗∗^*p* < 0.01.

Nuclear factor-κB signaling is involved in lung inflammation, and phosphorylation of NF-κB regulates the transcription of TNF-α, IL-6, and IL-1β. Thus, we tested the effects of H-SN1 on NF-κB pathway in lung tissue. As shown in **Figure 6**, western blot analysis revealed that LPS-induced phosphorylation of NF-κB and degradation of IκB-α were significantly prevented when pretreated with H-SN1. Furthermore, MAPKs have been demonstrated to participate in regulating the production of inflammatory cytokines. To fully explore the anti-inflammatory mechanism of H-SN1, the effects of H-SN1 on ERK pathway in lung tissues were also tested. Similarly, LPS administration induced an elevation in phosphorylation of ERK1/2, and treatment with H-SN1 decreased the phosphorylation of ERK1/2.

### *In Vitro* Experiment

#### H-SN1 Inhibits the Expression and Release of Inflammatory Cytokines in LPS-Stimulated RAW 264.7 Cells

**FIGURE 7:**
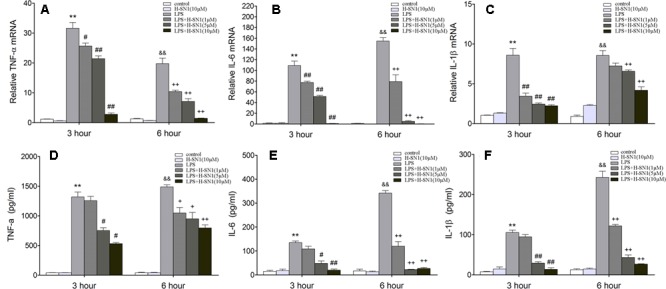
**Effect of H-SN1 on LPS-induced cytokines mRNA expression and production in RAW264.7 cells.** The mRNA expression of TNF-α **(A)**, IL-6 **(B)**, and IL-1β **(C)** was analyzed at 3 and 6 h post stimulation by RT-PCR. The production of TNF-α **(D)**, IL-6 **(E)**, and IL-1β **(F)** in supernatants was measured at 3 and 6 h post stimulation by enzyme-linked immunosorbent assay (ELISA). Data were presented as mean ± SEM (*n* = 4 in each group), ^∗∗^*p* < 0.01 and ^&&^*p* < 0.01 vs. Control group, ^#^*p* < 0.05, ^##^*p* < 0.01, ^+^*p* < 0.05, and ^++^*p* < 0.01 vs. LPS group.

To confirm the anti-inflammatory effect of H-SN1, we performed real-time RT-PCR and ELISA for TNF-α, IL-6, and IL-1β in LPS-stimulated RAW 264.7 cells. TNF-α, IL-6, and IL-1β mRNA expression levels in RAW 264.7 cells significantly increased at 3 and 6 h after stimulation with LPS (**Figures 7A–C**) and were inhibited by H-SN1 in a dose-dependent manner. Similarly, the cytokine levels in the supernatants obtained from LPS-treated RAW 264.7 cells were also elevated, and attenuated by H-SN1 treatment (**Figures 7D–F**).

#### H-SN1 Inhibits LPS-Induced NF-κB Activation and ERK Activation in RAW264.7 Cells

**FIGURE 8:**
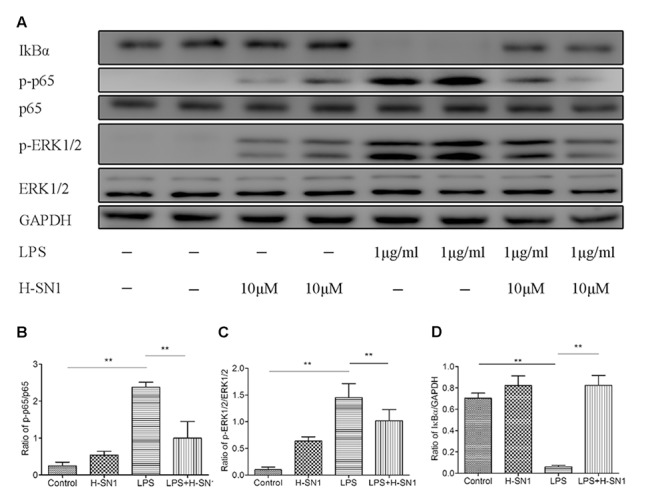
**Effect of H-SN1 on LPS-induced NF-κB and MAPK signaling activation in RAW264.7 cells.** The phosphorylation of NF-κBp65 and ERK1/2 proteins were analyzed at 30 min post stimulation by Western blot. **(A)** Representative immunoblots from three separate experiments with similar results are shown. **(B–D)** Relative protein levels were quantified by ImageJ software and expressed as optical density ratio. ^∗∗^*p* < 0.01. Raw data can be seen in **Supplementary Figure S8**.

Finally, to validate the findings from *in vivo* experiment, we tested the effects of H-SN1 on NF-κB pathway and ERK pathway in LPS-stimulated RAW 264.7 cells (**Figure 8**; Raw data can be seen in **Supplementary Figure S8**) with western blot analysis. In our current work, LPS dramatically enhanced NF-κB phosphorylation in RAW 264.7 cells. H-SN1 significantly inhibited NF-κB phosphorylation compared to LPS alone. In addition, the degradation of IκBα induced by LPS was attenuated after H-SN1 administration. Compared with the control group, LPS treatment resulted in phosphorylation of ERK1/2; this phosphorylation was decreased by H-SN1.

## Discussion

This is the first study that evaluated the anti-inflammatory effect of H-SN1, a peptide extracted from *H. cyanocinctus*, in an LPS-induced ALI model and LPS-stimulated RAW 264.7 cells. We found that treatment with H-SN1 significantly improved pulmonary edema, decreased vascular permeability, suppressed pro-inflammatory cytokine production, and lessened lung histopathological injury. In addition, *in vitro* experiments revealed that H-SN1 suppresses LPS-induced pro-inflammatory cytokine production in macrophages and that this suppression accompanies downregulation of ERK1/2 and NF-κB pathways. Taken together, our findings suggest that H-SN1 might be a potential new therapeutic agent for LPS-induced ALI by reducing the inflammatory response. The underlying mechanism was associated with the inhibition of ERK1/2 and NF-κB pathways.

Neutrophils are an important component of the inflammatory response that characterizes ALI. Several inflammatory mediators such as monocyte chemotactic protein 1 (MCP-1) and macrophage inflammatory protein 2 (MIP-2) are major chemotactic factors which play a pivotal role in recruiting neutrophils to the lung (Gupta et al., 1996; Takahashi et al., 2009). In endotoxemia-induced ALI, a large number of neutrophils infiltrate the lung tissue and migrate into the airways, leading to generation of reactive oxygen intermediates. One consequence of enhanced oxidative stress is the peroxidation of phospholipids, which causes plasma membrane damage and increased vascular permeability (Kunkel et al., 1991; Abraham, 2003; Nonas et al., 2006). MPO activity is a marker of parenchymal infiltration of neutrophils. The administration of H-SN1 clearly attenuated the recruitment of neutrophils in lung tissue from LPS-treated mice, indicated by two independent observations: decreased MPO activity in lung tissue and alleviated histopathologic changes.

Inflammatory mediators such as TNF-α, IL-6, and IL-1β play a critical role in the pathogenesis of ALI (Bhatia and Moochhala, 2004). IL-1β is one of cytokines which are at the upstream position of the inflammatory cytokine cascade for it can stimulate the production of many chemotactic cytokines such as MIP-1α, MCP-1, and IL-8 (Goodman et al., 2003). Similar to IL-1β, TNF-α can upregulate other pro-inflammatory cytokines and maintain inflammation, aggravating lung injury. Several studies have reported elevated levels of TNF-α in BAL fluid from ARDS patients (Hyers et al., 1991; Suter et al., 1992; Park et al., 2001). IL-6 is also an important pathogenic mediator of lung injury. Altmann et al. (2012) found serum IL-6 produced by circulating monocytes would stimulate alveolar macrophage production of CXCL1, leading to neutrophil recruitment and lung injury. Thus, numerous investigators have reported that inhibiting the overproduction of TNF-α, IL-6, and IL-1β cytokines can alleviate lung injury (Nonas et al., 2006; Hirano et al., 2015; Wang et al., 2016). In the present study, our *in vivo* and *in vitro* experiments both demonstrated that H-SN1 could significantly alleviate the LPS-induced increase in the expression and release of TNF-a, IL-1β, and IL-6. Similarly, in our previous work, H-SN1 treatment also reduced the levels of TNF-α, IL-6, and IL-1β transcripts in the inflamed colon (Zheng et al., 2016). Because H-SN1 is a TNFR1-binding peptide, we suspect that the anti-inflammatory effect of H-SN1 is partly due to inhibiting the TNF-α/TNFR1 interaction and thus the biological functions of TNF-α. Notably, since increasing evidences have shown lung epithelial and endothelial cells also play a key role in activating inflammation (Ochoa et al., 2010; Xie et al., 2016; Zhang et al., 2016; Kucukgul and Erdogan, 2017), we suspected that the decrease of inflammatory cytokines in the lung alveoli might be partially due to the effect of H-SN1 in suppressing inflammation in these cells. However, as the cell used in *in vitro* experiment was macrophage, which was one of antigen presenting cells that mediate the initiation of inflammatory reactions in lung injury (Mukhopadhyay et al., 2006), further study need to be done to clarify whether H-SN1 could also reduce inflammation in epithelium and endothelium, thus broadly suppress pulmonary inflammation.

It is well known that several signaling pathways participate in LPS-induced activation of macrophages and subsequent upregulation of pro-inflammatory cytokines such as TNF-α, IL-6, and IL-1β; the MAPK and NF-κB pathways play a pivotal role in this process (Choi et al., 2011; Xia et al., 2012). The activation of NF-κB is regulated via cytoplasmic sequestration of NF-κB by the inhibitor of κB (IκBs) family of proteins (Qiu et al., 2012). IκB-α, one isoform of IκBs, can sequester NF-κB in the cytoplasm. In our *in vitro* and *in vivo* models, we showed that LPS-induced increase in NF-κB p65 activation and degradation of IκBα were markedly inhibited by H-SN1. In addition, the MAPK42/44 (ERK1/2) pathway is also involved in the regulation of the inflammatory response of stimulated macrophages (Rao, 2001; Jiang et al., 2002; Schuh and Pahl, 2009). Similar to previous reports (Zhong et al., 2013; Zhang et al., 2014), LPS significantly induced the phosphorylation of ERK1/2, and this was significantly inhibited by pretreatment with H-SN1. Moreover, the total amount of ERK1/2 protein was unchanged, indicating that H-SN1 blocks the activation, but not the biosynthesis, of ERK1/2.

In summary, treatment with H-SN1 could attenuate LPS-induced ALI in mice by reducing inflammation. Our data indicate that H-SN1 attenuates the production of inflammatory cytokines via, at least in part, interfering with the ERK1/2 and NF-κB signaling pathways. Accordingly, H-SN1 might have potential applications as a supportive treatment for ALI. Further studies are needed to define the mechanisms that mediate the protective effects of H-SN1.

## Author Contributions

ZX, YL, and FZ developed the study concept and design. GW, JW, PL, and ST were involved in acquisition of the data. HJ, YZ, and AL performed statistical analysis. GW and AL wrote the manuscript. ZX and YL critically revised the manuscript. GW, JW, and PL contributed equally to this work.

## Conflict of Interest Statement

The authors declare that the research was conducted in the absence of any commercial or financial relationships that could be construed as a potential conflict of interest.

## Supplementary Material

FIGURE S1 **(A)** Hematoxylin and eosin (H&E) stain of representative lung from control mouse. **(B)** Hematoxylin and eosin (H&E) stain of representative lung from H-SN1 treated mouse. **(C)** Hematoxylin and eosin (H&E) stain of representative lung from LPS treated mouse. **(D)** Hematoxylin and eosin (H&E) stain of representative lung from LPS+400 μg/kg H-SN1 treated mouse.

FIGURE S8 **IκB-α: the degradation of IκB-α proteins were analyzed by Western blot.** p-ERK1/2: the phosphorylation of ERK1/2 proteins were analyzed by Western blot. p-p65: the phosphorylation of NF-κBp65 proteins were analyzed by Western blot.
