# Supplementary figures and images for: Hydrostatin-SN1, a Sea Snake-Derived Bioactive Peptide, Reduces Inflammation in a Mouse Model of Acute Lung Injury
Source: Front Pharmacol. 2017 May 5;8:246. doi: 10.3389/fphar.2017.00246 (PMC5418923; doi:10.3389/fphar.2017.00246)

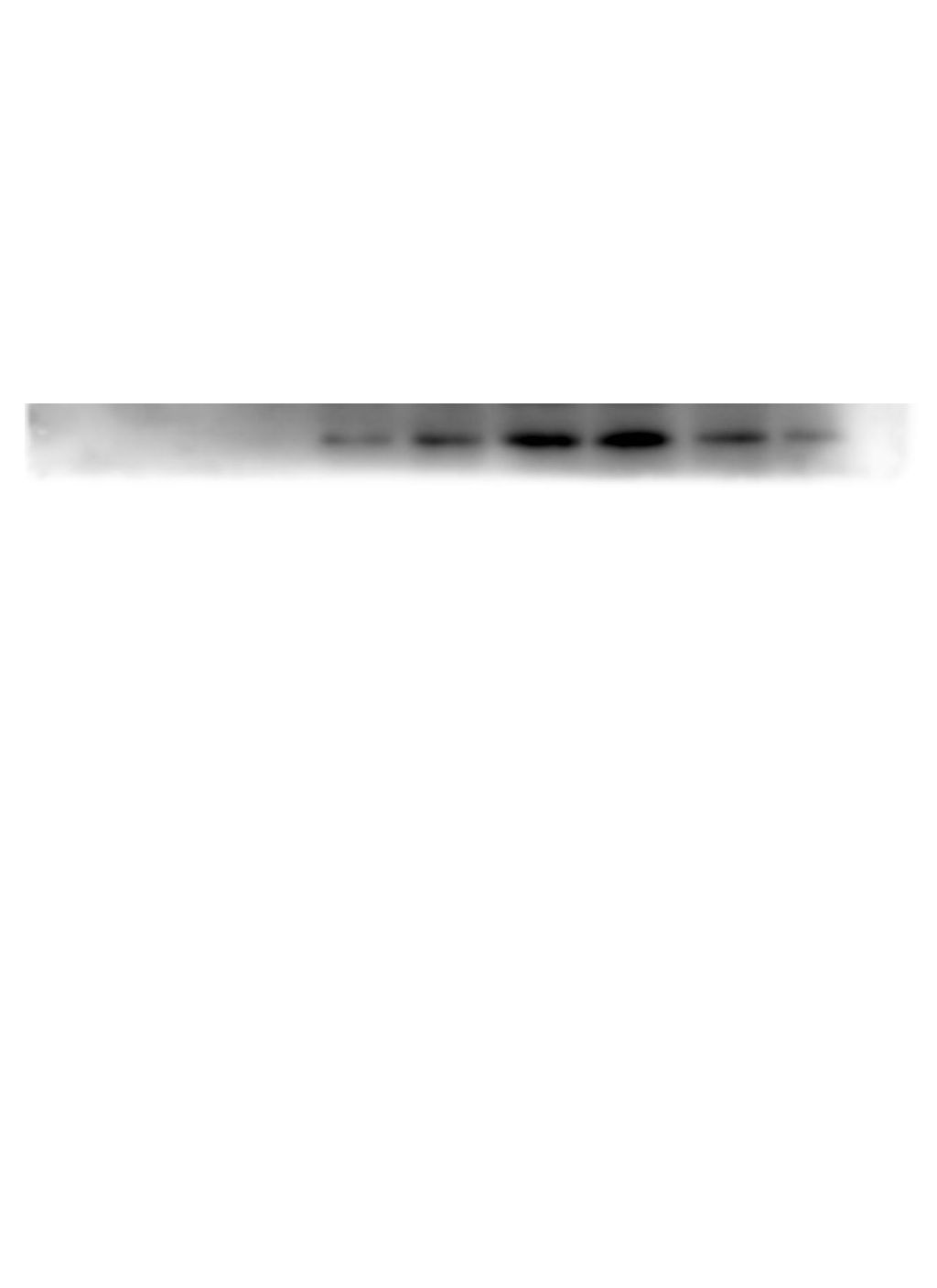

Supplement: FIGURE S1 — (A) Hematoxylin and eosin (H&E) stain of representative lung from control mouse. (B) Hematoxylin and eosin (H&E) stain of representative lung from H-SN1 treated mouse. (C) Hematoxylin and eosin (H&E) stain of representative lung from LPS treated mouse. (D) Hematoxylin and eosin (H&E) stain of representative lung from LPS+400 μg/kg H-SN1 treated mouse. [file Presentation_1.ZIP › Supplementary Raw data Fig8 p-p65.tif]

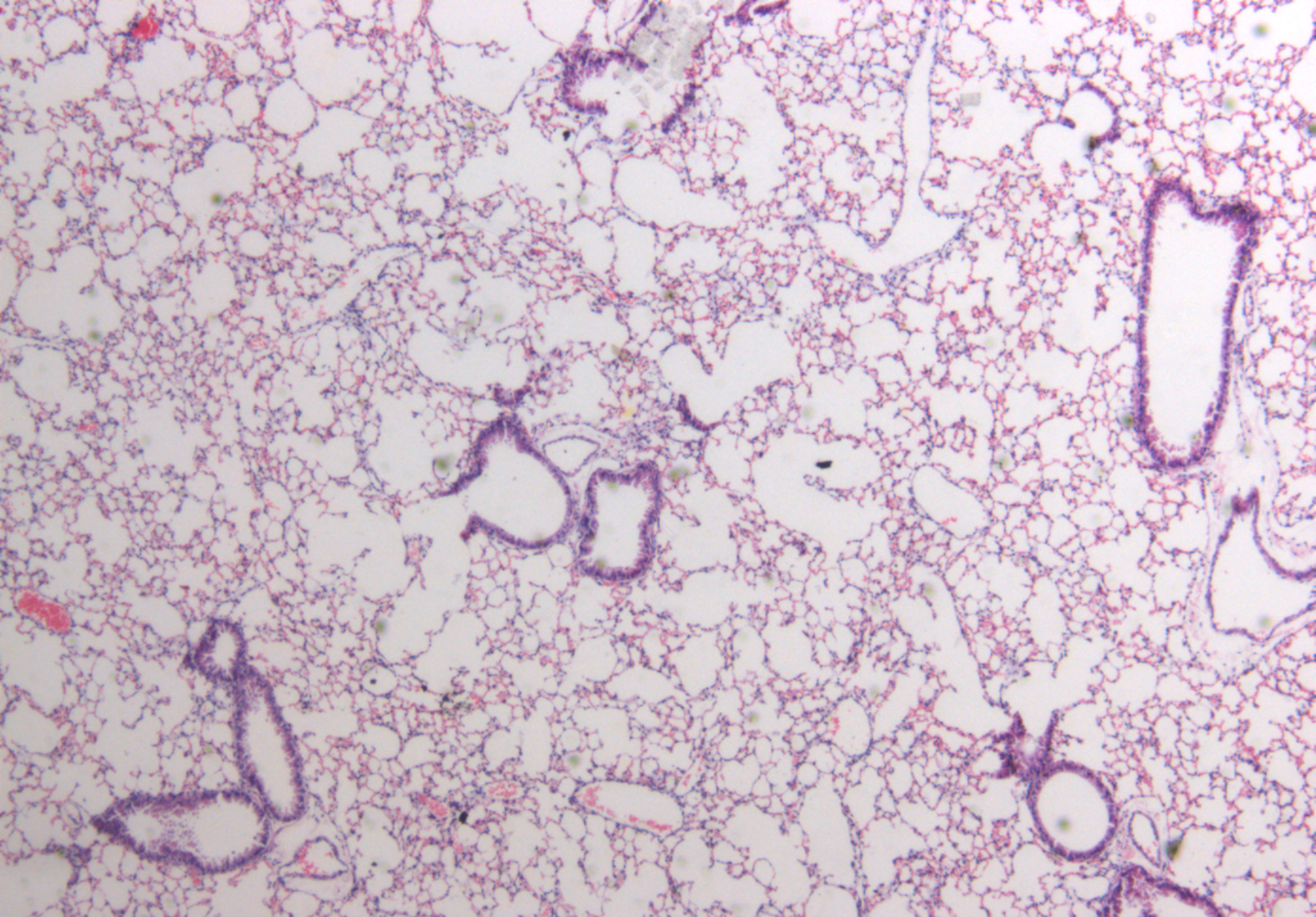

Supplement: FIGURE S1 — (A) Hematoxylin and eosin (H&E) stain of representative lung from control mouse. (B) Hematoxylin and eosin (H&E) stain of representative lung from H-SN1 treated mouse. (C) Hematoxylin and eosin (H&E) stain of representative lung from LPS treated mouse. (D) Hematoxylin and eosin (H&E) stain of representative lung from LPS+400 μg/kg H-SN1 treated mouse. [file Presentation_1.ZIP › Supplementary Raw data Fig 1 A.tif]

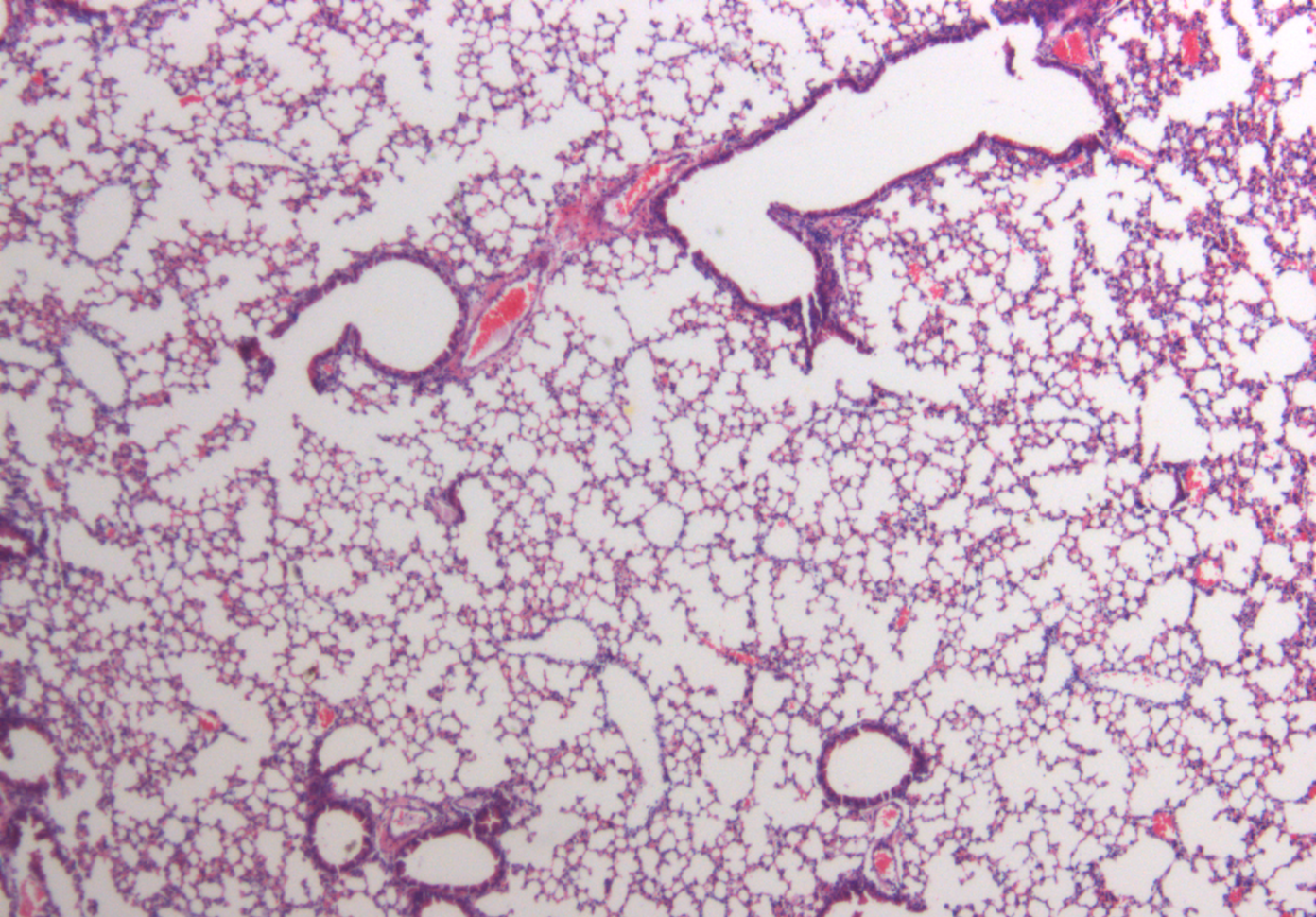

Supplement: FIGURE S1 — (A) Hematoxylin and eosin (H&E) stain of representative lung from control mouse. (B) Hematoxylin and eosin (H&E) stain of representative lung from H-SN1 treated mouse. (C) Hematoxylin and eosin (H&E) stain of representative lung from LPS treated mouse. (D) Hematoxylin and eosin (H&E) stain of representative lung from LPS+400 μg/kg H-SN1 treated mouse. [file Presentation_1.ZIP › Supplementary Raw data Fig 1 B.tif]

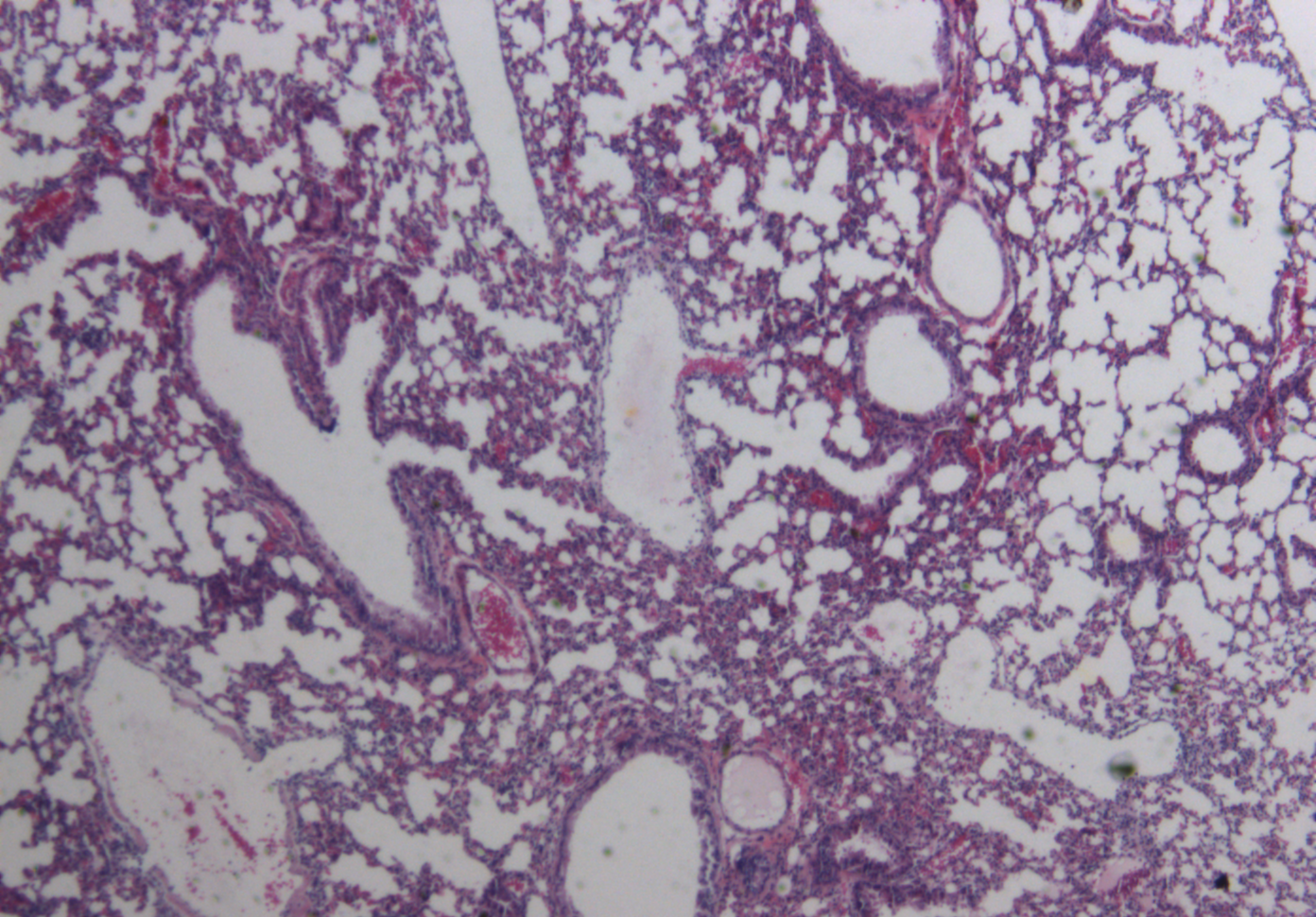

Supplement: FIGURE S1 — (A) Hematoxylin and eosin (H&E) stain of representative lung from control mouse. (B) Hematoxylin and eosin (H&E) stain of representative lung from H-SN1 treated mouse. (C) Hematoxylin and eosin (H&E) stain of representative lung from LPS treated mouse. (D) Hematoxylin and eosin (H&E) stain of representative lung from LPS+400 μg/kg H-SN1 treated mouse. [file Presentation_1.ZIP › Supplementary Raw data Fig 1 C .tif]

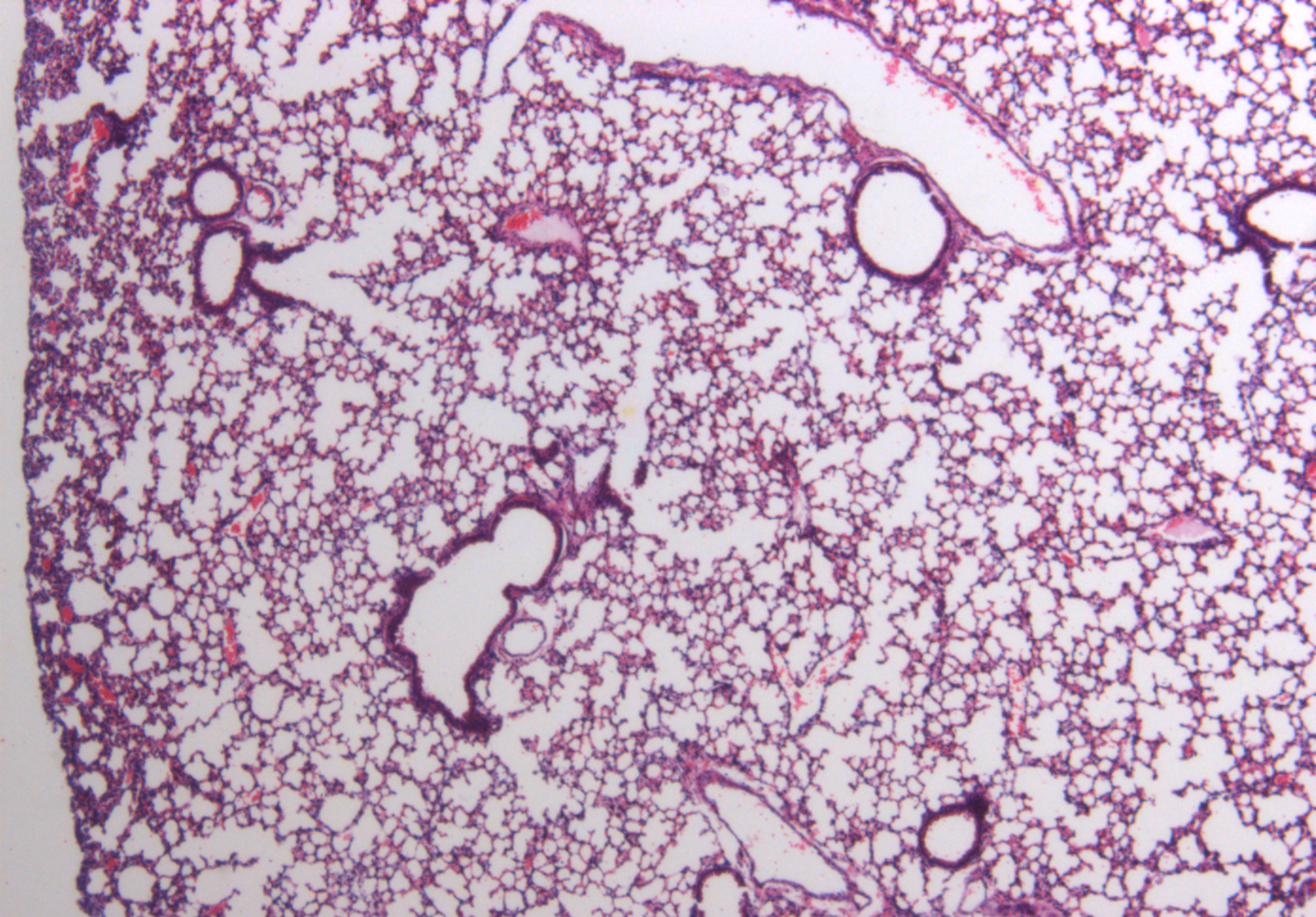

Supplement: FIGURE S1 — (A) Hematoxylin and eosin (H&E) stain of representative lung from control mouse. (B) Hematoxylin and eosin (H&E) stain of representative lung from H-SN1 treated mouse. (C) Hematoxylin and eosin (H&E) stain of representative lung from LPS treated mouse. (D) Hematoxylin and eosin (H&E) stain of representative lung from LPS+400 μg/kg H-SN1 treated mouse. [file Presentation_1.ZIP › Supplementary Raw data Fig 1 D.tif]

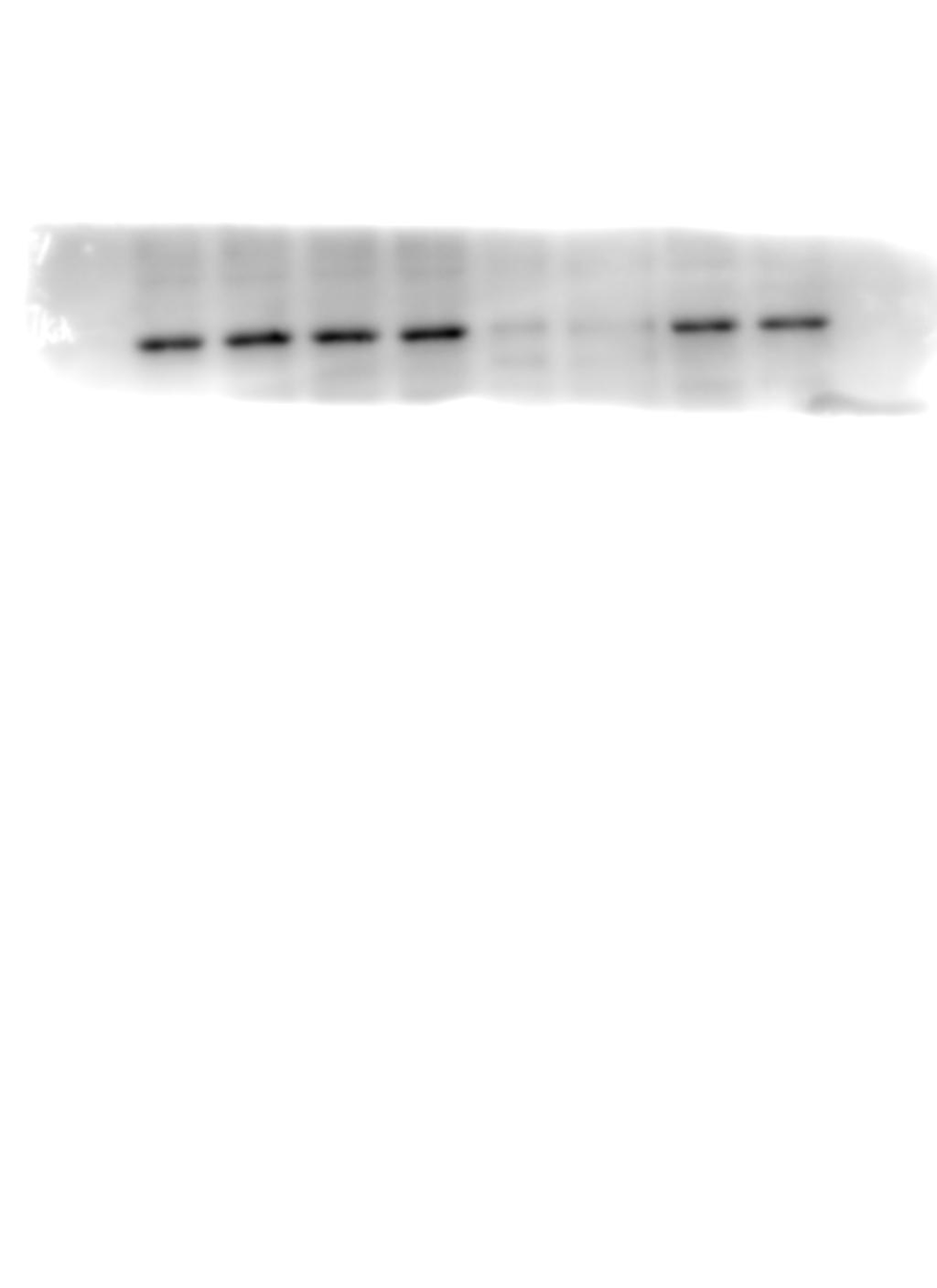

Supplement: FIGURE S1 — (A) Hematoxylin and eosin (H&E) stain of representative lung from control mouse. (B) Hematoxylin and eosin (H&E) stain of representative lung from H-SN1 treated mouse. (C) Hematoxylin and eosin (H&E) stain of representative lung from LPS treated mouse. (D) Hematoxylin and eosin (H&E) stain of representative lung from LPS+400 μg/kg H-SN1 treated mouse. [file Presentation_1.ZIP › Supplementary Raw data Fig8 IkBa .tif]

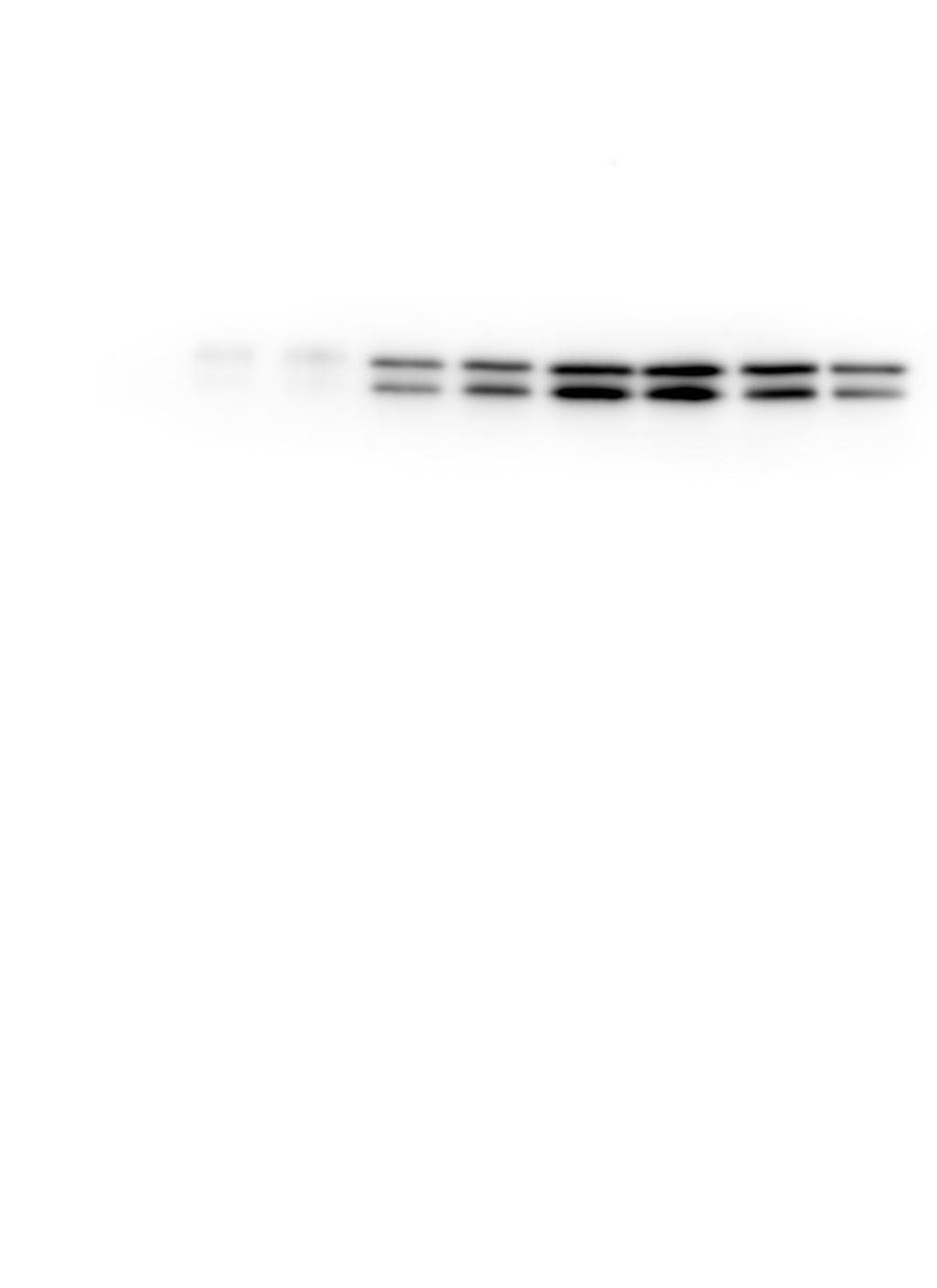

Supplement: FIGURE S1 — (A) Hematoxylin and eosin (H&E) stain of representative lung from control mouse. (B) Hematoxylin and eosin (H&E) stain of representative lung from H-SN1 treated mouse. (C) Hematoxylin and eosin (H&E) stain of representative lung from LPS treated mouse. (D) Hematoxylin and eosin (H&E) stain of representative lung from LPS+400 μg/kg H-SN1 treated mouse. [file Presentation_1.ZIP › Supplementary Raw data Fig8 p-ERK1_2 .tif]
